# Complete chloroplast genome characteristics of *Prunus triloba* Lindl

**DOI:** 10.1080/23802359.2019.1704657

**Published:** 2020-01-10

**Authors:** Chunyan Duan, Yehua Shen, Guifang Zhao

**Affiliations:** a Key Laboratory of Synthetic and Natural Functional Molecule Chemistry of Ministry of Education, College of Chemistry and Materials Science, Northwest University, Xi’an, China;; b Key Laboratory of Resources Biology and Biotechnology in Western China, Ministry of Education, School of Life Sciences, Northwest University, Xi’an, China;; c Agricultural College, Henan University of Science and Technology, Luoyang, China

**Keywords:** *Prunus triloba* Lindl, complete chloroplast genome, phylogenetic

## Abstract

*Prunus triloba* Lindl. is a small shrub species, with many varieties and a long history of cultivation. It has been widely used for landscaping and is grown as a traditional flowering tree species in northern China. In this study, we sequenced the *P. triloba* Lindl. chloroplast genome, which forms a circular structure comprising 158,455 bp, including a pair of inverted repeat regions (52,634 bp), a large single-copy region (86,386 bp), and a small single-copy region (19,028 bp). We annotated 131 genes, including 86 coding sequences, 8 rRNA sequences, and 37 tRNA sequences. Furthermore, a phylogenetic analysis revealed *P. triloba* Lindl. is closely related to *Prunus pedunculata*.

*Prunus triloba* Lindl., which belongs to the family Rosaceae, is a small shrub (approximately 2–3 m tall) (Editorial Committee of Flora of China of Chinese Academy of Sciences 1997) that is mainly distributed in several provinces in China, including Hebei, Shaanxi, Shandong, Yunnan, and Henan (Zhang et al. 2012). There are many *P. triloba* Lindl. varieties, with a long history of cultivation for landscaping and as a traditional flowering tree species in northern China (Chen 2001). Its characteristics include high adaptability, fast growth, and rapid germination (Cai et al. 2013; Liu et al. 2017).

Previous research regarding *P. triloba* Lindl. mainly focused on the development of plant tissue culture techniques as well as analyses of pollen tube germination, growth (Du et al. 2008), and biological characteristics (Zuo et al. 2007). In contrast, *P. triloba* Lindl. molecular mechanisms remain relatively uncharacterized. Chloroplast DNA (cpDNA), which includes important genes for energy conversion and photosynthetic activities, is present in the mesophyll cells of green plants. In addition to photosynthesis, mesophyll cells are also associated with the synthesis of chlorophyll, fatty acids, amino acids, starch, and other substances (Chen et al. 2004). In this study, we sequenced, assembled, annotated, and analyzed the *P. triloba* Lindl. chloroplast genome to elucidate the evolution and systematic taxonomy of *P. triloba* Lindl.

Fresh *P. triloba* Lindl. leaves were collected from plants growing in Yulin, Shaanxi province, China, in September 2018. Voucher specimens (20180915Yl06) were deposited in the herbarium of Yulin University, Shaanxi, China. The cpDNA was extracted from the fresh leaves according to a modified CTAB method (Doyle and Doyle 1987), after which the cpDNA was used for high-throughput sequencing with the Illumina HiSeq X Ten system. We used the *Prunus pedunculata* reference sequence (MG869261) for sequence assembly and annotation. We used the Geneious 8.0 program (Kearse et al. 2012) for annotating the complete *P. triloba* Lindl. chloroplast genome. An annotated cpDNA physical map was drawn using the OGDRAW online tool (Lohse et al. 2013). Moreover, cpDNA sequences were aligned with the MAFFT program (Kazutaka et al. 2002), whereas a phylogenetic tree was constructed according to the neighbor-joining method (1000 bootstrap replicates) with the MEGA 7.0 program. Finally, the complete chloroplast genome sequence was deposited in the GenBank database (MK790138).

The results revealed that the *P. triloba* Lindl. chloroplast genome forms a circular structure comprising 158,455 bp, with a pair of inverted repeat regions (52,634 bp), a large single-copy region (86,386 bp), and a small single-copy region (19,028 bp). We annotated 131 genes, which consisted of 86 coding sequences, 8 rRNAs, and 37 tRNAs.

A phylogenetic tree was constructed based on the following 10 complete chloroplast genomes (accession number in parentheses) (Figure 1): *P. triloba* Lindl. (MK790138), *P. mongolica* (KY073235), *P. pedunculata* (MG869261), *P. persica* (HQ336405), *P. kansuensis* (NC023956), *Malus baccata* (KX499859) (outgroup), *Ammopiptanthus mongolicus* (NC034742), *Ammopiptanthus nanus* (NC034743), *Platanus occidentalis* (DQ923116), and *P. humilis* (NC035880). The phylogenetic analysis indicated *P. triloba* Lindl. is closely related to *P. pedunculata*.

**Figure 1.**
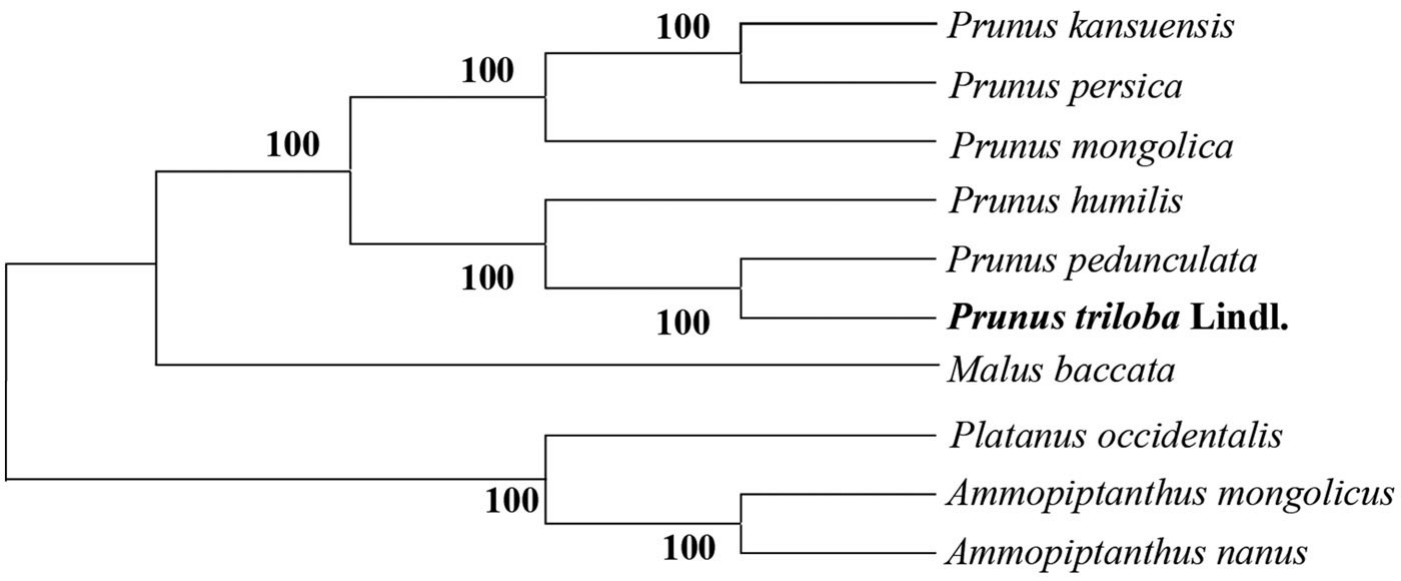
Phylogenetic tree based on the chloroplast genomes of 10 species. Accession numbers: *Prunus triloba* Lindl. (MK790138), *Prunus mongolica* (KY073235), *Prunus pedunculata* (MG869261), *Prunus persica* (HQ336405), *Prunus kansuensis* (NC023956), *Malus baccata* (KX499859), *Ammopiptanthus mongolicus* (NC034742), *Ammopiptanthus nanus* (NC034743), *Platanus occidentalis* (DQ923116), and *Prunus humilis* (NC035880).
